# Aberrant Drug-Related Behavior Monitoring

**DOI:** 10.31486/toj.20.0108

**Published:** 2020

**Authors:** Marianne Maumus, Renée Mancini, Daniel M. Zumsteg, Dileep K. Mandali

**Affiliations:** ^1^ Department of Hospital Medicine, Ochsner Clinic Foundation, New Orleans, LA; ^2^ The University of Queensland Faculty of Medicine, Ochsner Clinical School, New Orleans, LA; ^3^ Nursing Informatics, Center for Quality Excellence, Ochsner Clinic Foundation, New Orleans, LA

## TO THE EDITOR

**Figure 1.**
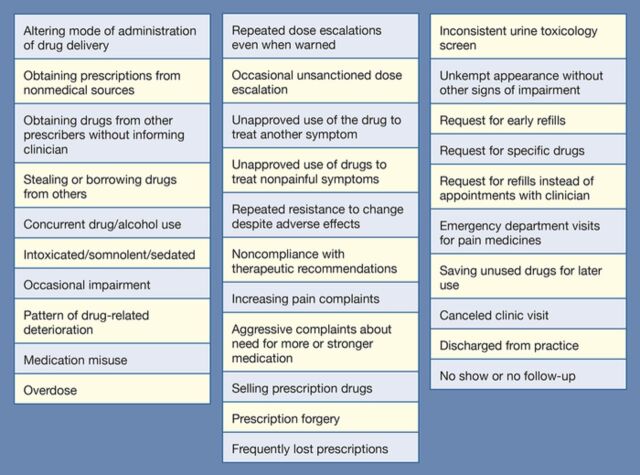
**Examples of aberrant behaviors.^1,4-7^**

A well-known comprehensive approach to the management of persistent pain is the Five A's of Pain Management: analgesia, activities of daily living, adverse effects, affect, and aberrant drug-related behaviors. Changes in the 5 A's during a course of opioid treatment signify complications of treatment, failure of therapy, and/or increased risk of progression toward opioid dependence and addictive disorders. Patients with opioid dependence and addictive disorders generally display one or more aberrant drug-related behaviors (Figure 1).^1-7^

The presence of aberrant behaviors in a patient who has been prescribed opioids is indicative of several possible problems, and a differential diagnosis should be explored.^8^ Possible etiologies include addiction, pseudo-addiction, another psychiatric disorder, personality disorder, chronic boredom, mild encephalopathy, and criminal intent to divert substances.^8^ Aberrant behaviors can also represent genuine undertreatment of pain.^8^ The behaviors are also present during withdrawal states. Aberrant behaviors are more common when associated with undertreatment in patients who have a history of prior substance abuse vs patients who are undertreated without a previous history of substance abuse.^8^ Understanding the patient-specific context associated with aberrant behaviors is therefore crucial in determining whether the behavioral pattern of a patient is a symptom of addiction, undertreatment, or one of many other causes.^8^

In the lead author's experience at Ochsner Health between 2002 and 2015, providers varied in their ability to recognize aberrant behavior and their willingness to document it. There was also a general lack of communication among specialties about aberrant behavior that led to overprescribing and subsequent underdiagnosing of iatrogenic addiction, which, in turn, precipitated an escalation of aberrant behavior, in particular, the verbal abuse of nurses and hospital physician providers. An objective means of monitoring and documenting aberrant behaviors across provider groups did not exist, and as a result, administrative staff and governing bodies of the institution could not track the patterns of aberrant behavior and prevent such behaviors.

A brief survey of physicians revealed that health care professionals may have preconceived notions about patients with different disease states and their pain sources, and aberrant behaviors may be misinterpreted in complex medical illnesses such as acquired immunodeficiency syndrome, sickle cell anemia, cancer, and former intravenous drug abuse.^8^ Physician perceptions of aberrant behaviors have a great degree of variability.^8^ Illegal behaviors such as the sale of prescription drugs, forging prescriptions, and altering a route of delivery were perceived to be more problematic than behaviors such as unkempt appearance, unsanctioned dose escalation, and hoarding of drugs. Because the interpretation of such behaviors is dependent on a provider's perception and experience—as well as the patient's clinical context—caution should be taken to avoid making spurious correlations.^8^ To promote a dispassionate interpretation of a patient's current state, the pattern of behavior in the setting of opiate use across multiple clinical environments needs to be established over the life course of a patient.

Categorizing and tracking various drug-taking behaviors is necessary to avoid personal bias and offhand judgments of patient behaviors.^8^ Tracking should be undertaken routinely with each admission and clinic appointment so that behaviors can be monitored during the course of care for which opioid treatment is indicated. Recognizing deterioration of patterns of behavior in various settings that signify progression on the pathway to addiction is important.^4,9^ Aberrant behaviors can signify red flags that have diagnostic significance, but they may also be common in nonaddicts and should be considered within the appropriate clinical context by the physician provider. However, nursing and therapy providers have the primary responsibility for documentation and communication of the presence of aberrant behaviors to physicians because they interact with the patient throughout the day.

Aberrant behavior monitoring in the hospital setting is important for several reasons:

1. Behavior monitoring is important to determine the success or failure of treatment, whether for patients receiving proper pain management with appropriate titration of opioids or for intentional withdrawal of opioid-dependent patients. In both clinical scenarios, behavior improves once the opioid dose becomes therapeutic in the former and when the physical dependence (or withdrawal state) is completed in the latter.
2. Early identification of potential opioid-related behavior can lead to proper opioid risk assessments to prevent the transition of chronic pain to opioid dependence and substance use disorder (SUD).^9^
3. Early identification of potential opioid-related behavior can lead to proper opioid risk assessments to prevent psychiatric disorders such as anxiety and depression.^4,9^
4. The provider's ability to recognize aberrant behaviors can lead to identification of undiagnosed SUD.
5. Aberrant behaviors must be stabilized to achieve proper medical management of acute illness and to improve adherence to medical and psychiatric treatment.
6. Aberrant behaviors must be stabilized for the safety and wellness of health care providers and nursing staff.
7. Aberrant behavior monitoring can help identify patients at high risk for diversion.
8. Aberrant behaviors are important diagnostic criteria for complications of opioid therapy and essential for evidence-based pain and opioid monitoring.

To remove bias, aberrant behaviors need to be objectified and monitored by an array of health care professionals, not just one provider. An honest exchange of information on monitoring adherence and documentation of behaviors over time would assist in decreasing the incidence of one provider simply labeling a patient as “drug-seeking.”^4^ Nurses, physical and occupational therapists, advanced practice practitioners, physicians, and psychiatrists should monitor and document aberrant behaviors in a team-based fashion. Combining this information with the patient's correct diagnosis allows the physician provider to align functional status, reports of subjective pain, morphine equivalent dose, nonopioid pain treatments, and aberrant behaviors with the expected course of pain treatment and proper adjustments of medication.

**Figure 2.**
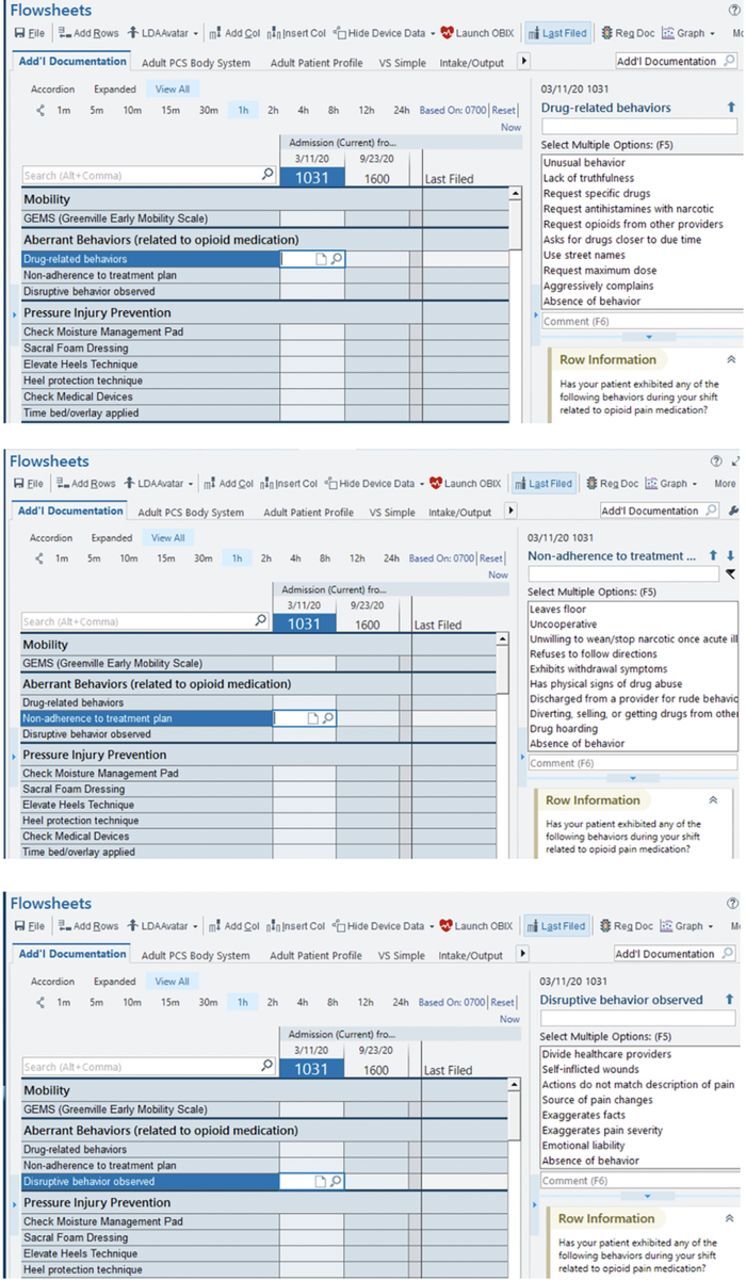
**Display of aberrant behaviors for nursing in the Epic electronic medical record at Ochsner Health. Aberrant behaviors have been subdivided into 3 categories for easy assessment and documentation: drug-related behaviors (top screen), nonadherence to treatment plan (middle screen), and disruptive behavior observed (bottom screen). Subcategories are examples of common observations that can trigger documentation.**

Figure 2 illustrates screens in the electronic medical record at Ochsner Health for documenting aberrant behaviors. Displays such as these can assist with proper documentation in a variety of hospital and clinic settings. Aberrant behaviors and other measures important to pain and addiction management could be graphed and monitored over time, combining both inpatient and outpatient settings. This monitoring would allow the effectiveness of various treatments to be displayed in an objective manner.

To properly interpret aberrant behaviors, providers must understand the following:

1. No single behavior equals addiction.^3^
2. Persistent pain creates stressors that interfere with adherence.^3^
3. Uncontrolled psychiatric illness can contribute to opioid misuse.^3^

Several screening tools have been developed to monitor for possible abuse and misuse of opioids. No single tool is available that can be uniformly applied to the current practice of pain management.^8^ At least 12 tools exist for clinical use, including the widely applied Opioid Risk Tool (ORT), the Current Opioid Misuse Measure, Pain Management Questionnaire, Screening Tool for Addiction Risk, and the Screener and Opioid Assessment for Patients with Pain. All of these tools use aberrant behaviors as an indicator for high opioid risk. These tools can be useful in specific populations but are not without limitations. While some are long questionnaires and more reliable, others are preferred for their brevity but are susceptible to deception.^8^ The ORT was chosen for use at Ochsner Health because of its brevity and ability to be applied as a systemwide intracommunication device about risk and to stimulate more providers to complete risk assessments. The ORT, however, is not a complete measure of risk. The presence of aberrant behaviors is a component of an overall diagnosis, but the context, pattern with disease activity, and opioid use need to be considered to gain a holistic understanding of the patient's state.

Long-term opioid therapy for chronic pain is associated with adverse side effects, as well as the potential for misuse and abuse, illicit drug use, and diversion.^8^ Given the subjective nature of pain—and its complex picture affected by psychiatric, neurologic, and social conditions—all diagnostic methods and capabilities need to be made available to providers in a transparent and unbiased manner. This information will allow providers to effectively track patient behaviors over the course of opioid treatment, to better communicate patient care among specialties, and to thereby mitigate the risk of overprescribing opioids or promoting aberrant behavior. Ultimately, physicians and their health care systems are responsible for recognizing the potential for and occurrence of opioid abuse and to develop a means of predictive opioid abuse assessment, determination, and treatment.^8^
